# Does F_4_TCNQ Adsorption on Cu(111) Form a 2D-MOF?

**DOI:** 10.1021/acs.jpcc.3c04927

**Published:** 2023-10-12

**Authors:** Pengcheng Ding, Mona Braim, A. L. Hobson, L. A. Rochford, P. T. P. Ryan, D. A. Duncan, T.-L. Lee, H. Hussain, G. Costantini, Miao Yu, D. P. Woodruff

**Affiliations:** † Department of Physics, University of Warwick, Coventry CV4 7AL, U.K.; ‡ Laboratory for Space Environment and Physical Sciences, School of Chemistry and Chemical Engineering, Harbin Institute of Technology, Harbin 150001, China; § Diamond Light Source, Harwell Science and Innovation Campus, Didcot, Oxford OX11 0DE, U.K.; ∥ Department of Materials, Imperial College London, London SW7 2AZ, U.K.; ⊥ Department of Chemistry, University of Warwick, Coventry CV4 7AL, U.K.; # School of Chemistry, University of Birmingham, Birmingham B15 2TT, U.K.

## Abstract

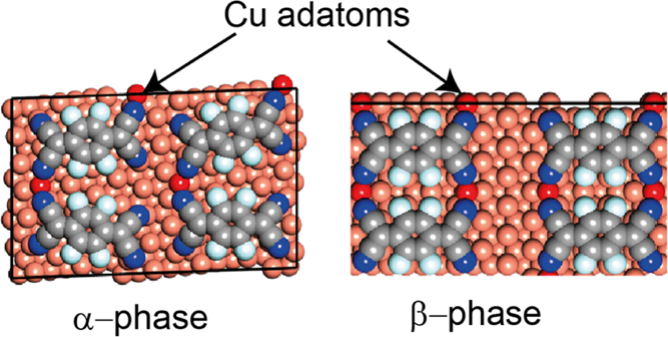

The results of a quantitative experimental structural investigation of the adsorption phases formed by 2,3,5,6-tetrafluoro-7,7′,8,8′-tetracyanoquinodimethane (F_4_TCNQ) on Cu(111) are reported. A particular objective was to establish whether Cu adatoms are incorporated into the molecular overlayer. A combination of normal incidence X-ray standing waves, low-energy electron diffraction, scanning tunneling microscopy, and X-ray photoelectron spectroscopy measurements, complemented by dispersion-inclusive density functional theory calculations, demonstrates that F_4_TCNQ on Cu(111) does cause Cu adatoms to be incorporated into the overlayer to form a two-dimensional metal–organic framework (2D-MOF). This conclusion is shown to be consistent with the behavior of F_4_TCNQ adsorption on other coinage metal surfaces, despite an earlier report concluding that the adsorption structure on Cu(111) is consistent with the absence of any substrate reconstruction.

## Introduction

The electronic properties of devices based on organic semiconductors can be strongly influenced by the nature of metal–organic interfaces at conductive electrodes, with electron acceptor and donor molecules modifying the charge injection barrier at such interfaces. For example, the electron acceptor molecule 7,7,8,8-tetracyanoquinodimethane (TCNQ) and its more strongly electron-accepting fully fluorinated variant, 2,3,5,6-tetrafluoro-7,7′,8,8′-tetracyanoquinodimethane (F_4_TCNQ), have been used in photovoltaic devices,^1,2^ organic light-emitting diodes,^3,4^ and field-effect transistors.^5,6^ This has motivated a number of surface science studies of these molecules on coinage metal surfaces, particularly with a (111) orientation (e.g., see refs (7−16)). Spectroscopic studies clearly demonstrate charge transfer from the metal surface, leading to rehybridization of the intramolecular bonding that relaxes the rigidity of the planar gas-phase molecule.^17^ The results of many density functional theory (DFT) calculations predicted that these molecules, when adsorbed on coinage metal (111) surfaces, adopt a symmetrical inverted bowl or umbrella conformation, with the cyano N atoms bonding to the surface, while the central quinoid ring is up to ∼1.5 Å higher above the surface. An implicit assumption in these earlier computational studies was that these molecular electron acceptors form a purely organic layer on an unreconstructed metal surface.

However, more recent studies using quantitative experimental structural techniques (normal incidence X-ray standing waves—NIXSW^18^ and surface X-ray diffraction—SXRD)^19^ have shown that there are three systems, namely, F_4_TCNQ on Au(111),^20,21^ TCNQ on Ag(111),^22,23^ and F_4_TCNQ on Ag(100),^24^ in which adsorption leads to the incorporation of metal adatoms from the substrate to form two-dimensional metal–organic frameworks (2D-MOFs) on the surface. This structural modification can cause significant changes in the surface dipoles and thus in the electronic structure of the interface; so, understanding the conditions that lead to this effect has wide relevance. While this surface reconstruction has been observed for F_4_TCNQ on Au and Ag surfaces, an early study of F_4_TCNQ adsorption on Cu(111) concluded that NIXSW and DFT results were compatible with adsorption on an unreconstructed surface.^8^

It is difficult to understand why the behavior of F_4_TCNQ adsorption on Cu(111) should not show adsorbate-induced metal adatom incorporation. DFT calculations for F_4_TCNQ on Au(111), Ag(111), and Cu(111) (but neglecting the influence of any possible adsorbate-induced reconstruction) show that the bonding strength is weakest on Au surfaces, stronger on Ag surfaces, and strongest on Cu surfaces,^25,26^ perhaps indicating that there is a greater probability of adsorbate-induced surface reconstruction on Cu(111). Notice, too, that the cohesive energy of Cu, a parameter that is related to the energy cost of adatom extraction, is intermediate to that of Au and Ag, so Cu is not an outlier in this regard (e.g., ref (27)). Thus, since adatom incorporation is energetically favored on both Au and Ag, there is not a clear rationale for why it would not be favored on Cu as well. Here, we report the results of a new combined experimental and theoretical study of the Cu(111)-F_4_TCNQ system to re-examine this apparent dilemma.

Our approach is initially to characterize carefully the long-range order of the adsorption phases of F_4_TCNQ on Cu(111) under different preparation conditions using scanning tunneling microscopy (STM) and low-energy electron diffraction (LEED). Soft X-ray photoelectron spectroscopy (SXPS) measurements were taken to identify the different C 1s chemically shifted components. Normal incidence X-ray standing waves (NIXSW)^18^ at the Cu(111) Bragg reflection condition were then made to determine the adsorption heights of the elementally and chemically inequivalent C, N, and F atoms above the surface. Finally, we performed dispersion-inclusive DFT calculations of alternative structural models to identify the lowest energy structures and to compare the predicted atomic heights of the constituent atoms with the NIXSW experimentally determined values. While the earlier investigation of this system^8^ also used a combination of NIXSW measurements and DFT calculations, it failed to identify which adsorption phase was measured and used a significantly lower spectral resolution in the NIXSW data than in our new experiments (such that chemically distinct C atoms could not be distinguished). Furthermore, the DFT calculations in this earlier study took no account of dispersion forces and failed to consider the possible impact of any surface reconstruction. We also note that the reported values of the NIXSW coherent fractions were so low as to suggest that the surface phase investigated may not have comprised a single molecular layer.^28^

## Methods

### Experimental Details

Experimental characterization of the adsorption phases of F_4_TCNQ on Cu(111) was performed using STM and low-current (microchannel plate) low-energy electron diffraction (MCP-LEED) in an ultrahigh vacuum (UHV) surface science chamber at the University of Warwick and by MCP-LEED and SXPS in the UHV end-station of beamline I09 of the Diamond Light Source.^29^ The Cu(111) sample was cleaned in situ by cycles of 1.0 keV Ar^+^ ion bombardment and annealing at 500 °C in both chambers. Single molecular monolayer structures were prepared by vacuum deposition from evaporation sources installed in the chambers. NIXSW experimental data were collected from F_4_TCNQ on Cu(111) by measuring the C 1s, N 1s, and F 1s photoelectron spectra as the incident photon energy was stepped through the (111) Bragg reflection, very close to normal incidence to the (111) surface, around a photon energy of 2974 eV. Comparisons of the relative intensity of the component peaks as a function of photon energy with standard XSW formulas, taking account of the backward–forward asymmetry of the angular dependence of the photoemission, allowed the optimum values of the coherent fraction and coherent positions to be determined.^18^

### Computational Details

DFT calculations were performed using the planewave pseudopotential package Quantum Espresso (QE).^30^ The interaction between electrons and ion cores was described by the projected augmented wave method with a plane-wave cutoff energy of 450 eV. The exchange and correlation effects were treated by the Perdew–Burke–Ernzerhof^31^ exchange–correlation density functional. A correction for the influence of van der Waals (vdW) forces was calculated with the zero damping DFT-D3 method of Grimme.^32^ The Cu(111) surface was represented by a repeated slab of three Cu layers separated by a 15 Å vacuum gap; only the coordinates of the bottom layer of the slab were constrained to bulk values. The Brillouin zone was sampled through the Monkhorst–Pack scheme. The models for the two ordered overlayer phases, the “α phase” and the “β phase”, were built in extended commensurate supercells of  and , respectively, to comply with the periodic boundary conditions, and the *k*-point mesh was set to be 1 × 2 × 1. All structures were optimized until the residual forces were smaller than 0.02 eV/Å.

## Results and Discussion

### Experimental Surface Characterization

**Figure 1:**
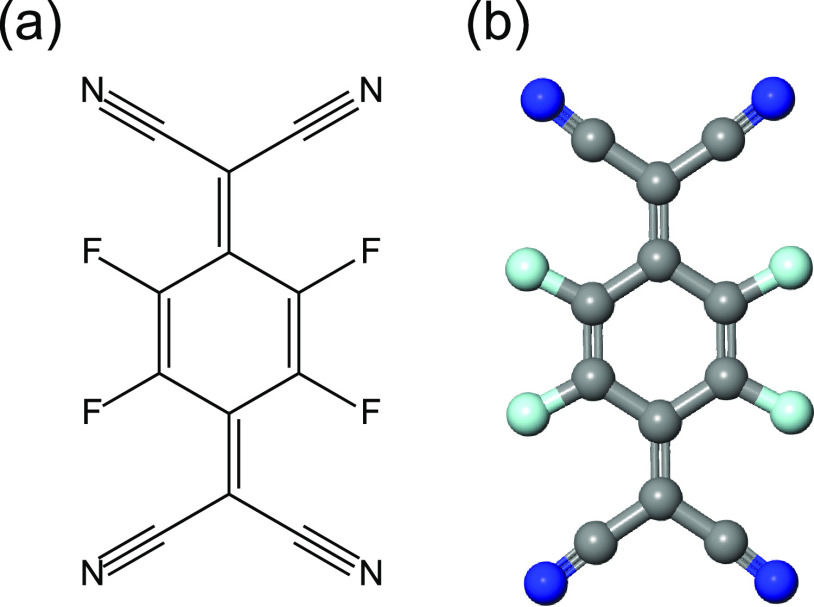
Structural formula of F_4_TCNQ (a) and a ball-and-stick model (b) showing the atom coloring scheme used in structural models displayed later in this paper.

**Figure 2:**
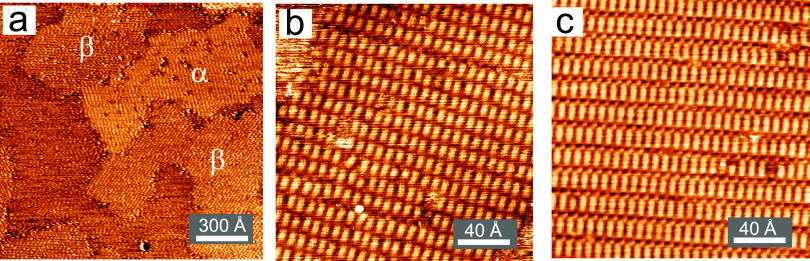
STM images of the ordered phases of F_4_TCNQ on Cu(111). (a) A large area with coexisting areas of the α and β phases. (b, c) Higher magnification images of, respectively, the α and β phases. Tunneling conditions, sample bias, and current: (a) −1.1 V, 0.3 nA; (b) 0.3 V, 0.4 nA; and (c) −1.0 V, 0.3 nA.

**Figure 3:**
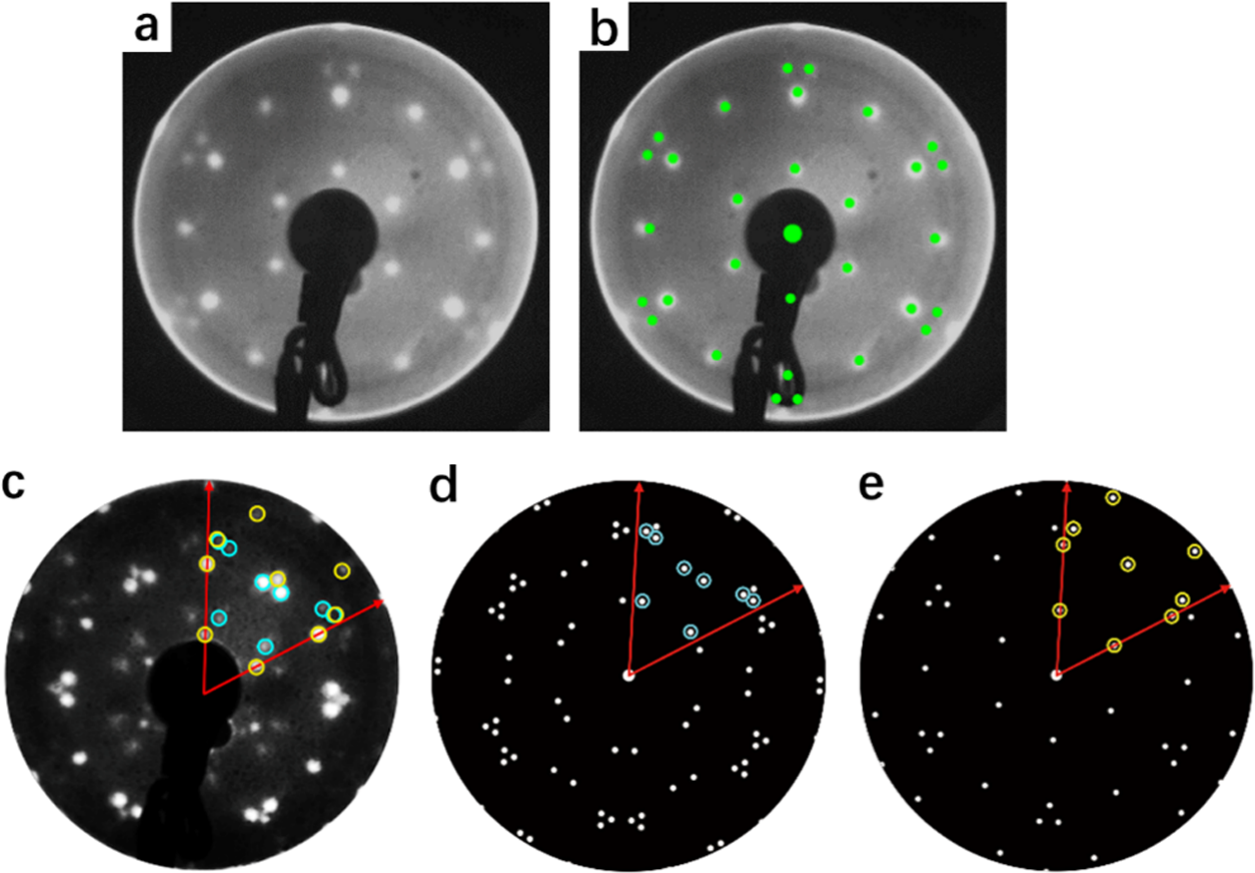
(a) LEED pattern obtained from a surface showing only the β phase recorded at an electron energy of 15.0 eV. (b) Same LEED pattern with superimposed green spots corresponding to the LEED pattern predicted for a  phase according to the LEEDpat program.^33^ (c) LEED pattern from a RT preparation of the F_4_TCNQ-covered Cu(111) surface at an electron energy of 15.0 eV, while panels (d) and (e) show LEEDpat simulations for the /α and /β phases, respectively. Superimposed on the experimental LEED pattern (c) are the predicted locations of diffracted beams from the  (green) and  (yellow) phases.

Deposition of F_4_TCNQ (Figure 1) on the Cu(111) surface at room temperature led to the coexistence of two ordered phases that could be observed in STM images. The large-area STM image in Figure 2a shows coexisting islands of the two molecular phases, which we refer to as the “α” and “β” phases. The α phase has a brighter contrast and more defects in the molecular islands than the β phase. Smaller-area STM images of single-phase regions in Figure 2b,c also show the molecules to be less well-ordered in the α phase than in the β phase. Exploration of the effect of different annealing conditions revealed that annealing a sample prepared at room temperature to a nominal temperature of 187 °C led to almost complete transformation of the α phase to the β phase (see the LEED patterns obtained at successively higher annealing temperatures in Figure S1), although no conditions could be identified under which only the α phase was formed. The corresponding matrixes for the α and β phases, extracted from the measured periodicity of the STM images, are  and , respectively. The half-integer matrix values raise the question of whether these adsorption phases are commensurate or incommensurate. In a truly commensurate overlayer, the periodicity of the overlayer-plus-surface must be described by a matrix comprising only integer values. However, STM typically samples only the periodicity of the outermost atoms, which may not reflect the periodicity of the complete overlayer-plus-underlying surface. LEED, on the other hand, samples the periodicity of the outermost few atomic layers, potentially offering a way to determine the true periodicity of the complete surface. Nevertheless, as shown in Figure 3a, a LEED pattern recorded from a surface showing only the β phase in the STM corresponds exactly to what would be expected from a surface showing  periodicity (green spots in Figure 3b). Moreover, the LEED pattern recorded from the as-prepared surface at room temperature (Figure 3c) can be fully reconciled with a superposition of the predicted LEED patterns for coexisting  and  phases, as shown in Figure 3d,e. The implication is that while the true commensurate phases may be described by larger  and  (or ) matrices, the additional diffracted beams that would be associated with these larger meshes due to multiple scattering between the overlayer and substrate (together with adsorbate-induced distortion of the outermost Cu(111) atomic layers) might be too weak to be observed.

While no preparation conditions were identified that led to only the α-phase being present, annealing a mixed-phase surface did lead to the removal of the α phase, as was observed for depositing onto a sample at an elevated temperature; LEED patterns from the annealed surface were consistent with only the β phase (Figure 3a). This transformation of the LEED pattern to the β phase upon annealing (Figure S1) was reproduced in both the University of Warwick and Diamond experiments, although the apparent temperature (∼200 °C) associated with this transition differed significantly in the two experimental chambers, presumably due to the difficulty in measuring the true sample temperature.

**Figure 4:**
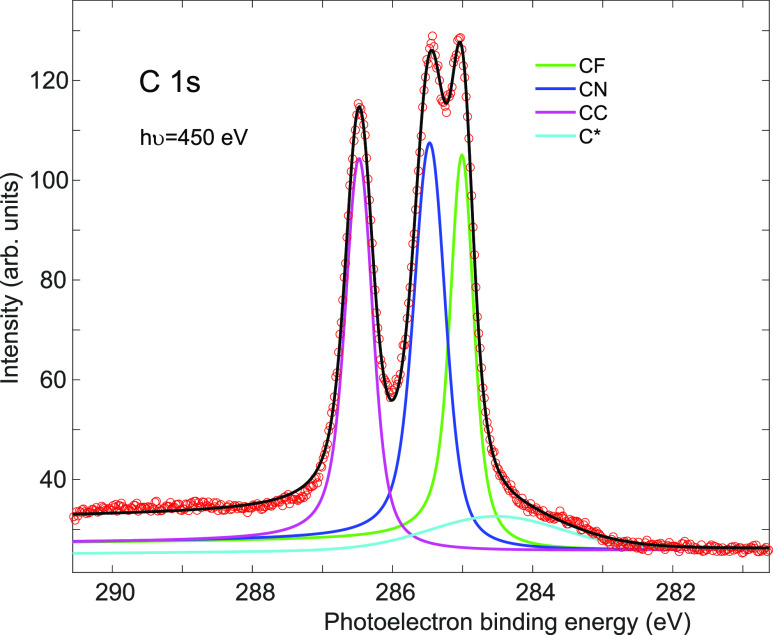
C 1s SXP spectrum from a mixed phase of F_4_TCNQ on Cu(111). Also shown are the different spectral components used to fit the experimental data.

These adsorption phases were also characterized by soft X-ray photoelectron spectroscopy (SXPS). The C 1s spectrum recorded from a weakly annealed preparation of the F_4_TCNQ adsorption structure, yielding a LEED pattern consistent with the coexistence of the α phase and the β phase (Figure 3c), is shown in Figure 4. This spectrum shows three main chemically shifted components assigned to the CF-, CN-, and CC-bonded C atoms; the relative binding energies of these components are consistent with electron transfer from the metal to the F_4_TCNQ molecule, as reported for adsorption on Au(111)^21^ and Ag(100).^24^ In addition, however, there is also a weaker fourth C 1s component at the lowest binding energy (labeled C* in Figure 3c) that we attribute to a dissociated fragment, possibly atomic C. The N 1s and F 1s spectra from this surface are shown in Figure S2 of the Supporting Information. These show a single N 1s peak, which indicates that all N atoms occupy closely similar chemical environments. However, annealing of the surface to a higher temperature needed to achieve a LEED pattern characteristic of only the β phase led to pronounced changes in the SXP spectra, with additional chemically shifted components in the C 1s spectra and a second F 1s peak (Figure S3). The F 1s peak at this binding energy has been identified, in a study of the synthesis of rare-earth metal fluorides, due to a metal fluoride.^34^ Evidently, annealing leads to some partial decomposition and reaction of the adsorbed F_4_TCNQ, apparently leading to some F bonding to the metal surface. Reinspection of the STM images obtained after annealing indicates that while well-ordered β-phase regions account for the characteristic  LEED pattern, disordered regions, which grow with increasingly higher temperature annealing (see Figure S4), also exist; we attribute these to the reacted or decomposed material.

### NIXSW Structure Determination

In light of the SXPS evidence that the formation of the β phase is accompanied by dissociated species in disordered parts of the surface, our NIXSW studies focused on the as-prepared surface after only slight annealing, which LEED indicates to comprise mostly α phase but with some fraction of the β phase. NIXSW relative photoemission intensity profiles for photon energies around the normal incidence (111) Bragg condition were extracted for the N, F, and chemically distinct C components from these spectra. NIXSW photoemission-derived absorption profiles can be fitted (taking account of nondipole effects in the angular dependence to the photoemission^18^) uniquely by two parameters: the coherent fraction, *f*, and the coherent position, *p*. In the idealized situation of an absorbing atom occupying a single well-defined site with no static or dynamic disorder (*f* = 1.0), the coherent position (expressed in units of the Bragg plane spacing, *d*, 2.08 Å for Cu(111)) can be related to the height of this absorber site above the Bragg diffraction planes, *D* = (*p* + *n*)*d*, where *n* is an integer (usually 0 or 1) chosen to ensure that the implied interatomic distances are physically reasonable.^18^ The coherent fraction is commonly regarded as an order parameter, including the effects of atomic vibrational amplitudes, and some static disorders can lead to *f* values as low as ∼0.70,^28^ but much lower values can only be attributed to the contributions of at least two distinctly different absorber heights.^28^Figure S5 shows the experimental NIXSW absorption profiles and best fits of the data. Table 1 shows the *f* and *D* values obtained from fitting of the NIXSW data.

**Table 1:** Summary of the Values of the Structural Parameters Extracted from the NIXSW Measurements from the Room Temperature Preparation of F_4_TCNQ on Cu(111)^a^

component	*F*	*D* (Å)
CF	0.87(10)	3.33(5)
CC	0.64(10)	3.32(5)
CN	0.60(10)	3.15(5)
N	0.43(10)	3.11(5)
F	0.43(10)	3.31(5)

a Precision estimates in the final decimal place are shown in parentheses.

The high (0.87) coherent fraction for the CF atoms clearly indicates that all of these atoms have essentially the same height above the surface such that the quinone ring is parallel to the surface. By contrast, the much lower coherent fraction for the N atoms (0.43) indicates that there must be at least two different N atom heights in the surface structure. These different N heights would lead to some difference in height of the C atoms bonded to the N atoms (the “CN” atoms), which could account for the reduced coherent fraction of the CN atoms. In our previous NIXSW studies demonstrating the presence of metal adatoms in the molecular overlayer, a characteristic “signature” of this effect was a low coherent fraction of the N atoms, resulting from a twisting of the cyano end groups. This twisting was attributed to there being a mixture of some N atoms bonding to adatoms, while others are bonded to the underlying substrate. The results of Table 1 are consistent with this same picture.

The origin of the low coherent fraction for the F atoms is unclear, but this same effect was found in NIXSW studies of F_4_TCNQ on Au(111)^21^ and Ag(100).^24^ Enhanced vibrational amplitudes may be expected for these atoms but are unlikely to be sufficiently large to account for such low coherent fractions.^28^ The significant molecular modification resulting from heating leads to the creation of a chemically distinct F species, as witnessed by the second F 1s SXP peak (Figure S3), but a more minor modification could, perhaps, lead to partial creation of a second species (and local structure) with no significant chemical shift.

### Structure Characterization by Density Functional Theory

**Figure 5:**
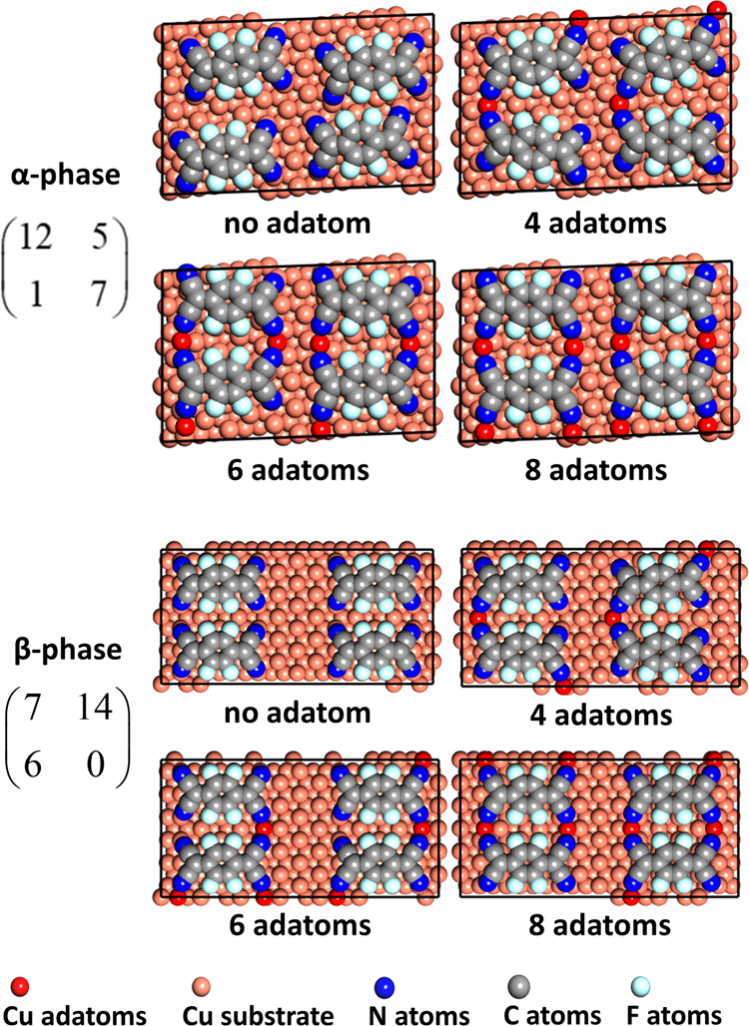
Structural models optimized in the DFT calculations. Each model contains 4 F_4_TCNQ molecules per unit mesh together with a variable number of Cu adatoms. The coloring of the atoms in the molecules is shown in Figure 1. Cu adatoms are shown a darker shade of red than the substrate Cu atoms.

In order to understand and determine the structure more completely, dispersion-inclusive DFT calculations were performed to determine the minimum energy configurations and to compare the experimental NIXSW structural parameters to those of the energetically preferred models. Of course, the fact that the experimental data correspond to a state in which two different ordered phases coexist means no truly unique solution can be expected, while the large unit meshes of the two phases also introduce significant complexity. Furthermore, the DFT slab calculations can only be performed on truly commensurate structural models, so superstructure models with all matrix components integral must be used. Specifically, calculations were performed using a  mesh for the α phase and a  mesh for the β phase. The β phase could be consistent with a commensurate mesh, but the larger mesh we have selected has almost the same area as that of the α-phase rendering the results of the DFT calculations for the two phases more comparable. A range of possible structural models for these two similarly sized meshes considered is shown in Figure 5.

The STM images of Figure 2b,c provide a clear indication of the molecular packing but not, of course, of the exact molecule–substrate lateral registry or of the presence (and location) or absence of Cu adatoms. These considerations led to calculations being performed for 4 basic structural models for each phase, as shown in Figure 5. These models differ primarily in the number of Cu adatoms per unit mesh but are not exhaustive in that other arrangements of the adatoms may be considered for the models having less than one Cu adatom per adsorbed F_4_TCNQ molecule. The DFT calculations yield two important results for each of these structural models, namely, the adsorption energy per molecule and the exact structural parameter values that can be converted into the values of the NIXSW coherent fraction and position that would be obtained from each structure.

The adsorption energy per molecule is defined as
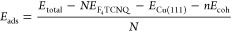
where *E*_total_ is the total energy of the complete adsorption structure in the unit mesh, *N* is the number of F_4_TCNQ molecules in the unit mesh, *E*_F_4_TCNQ_ is the energy of a free F_4_TCNQ molecule, *n* is the number of Cu adatoms in the unit mesh, and *E*_coh_ is the cohesive energy of bulk Cu. This last term corrects for the different numbers of total Cu atoms in each structure.

The main results of the DFT calculations are summarized in Tables 2 and 3. These show the predicted NIXSW parameters for the optimized structure of each model, together with the associated adsorption energy per molecule. Notice that the predicted coherent fractions take account of only the effect of different heights of atoms of the same chemical character in the static structure. To compare these predicted values with those found in the experiments, one must reduce them by between 5 and 30% to take account of possible static and dynamic disorders.^28^ The calculated adsorption energies clearly favor the presence of some Cu adatoms over the no adatom model with just one exception, the 8-adatom model of the α phase, which is slightly less energetically favorable than the no adatom model. Furthermore, although the predicted adsorption height for the N atoms for the no adatom model of 2.12 Å is closely similar to the other published computed values for this structure using van der Waals-corrected DFT calculations (2.21 Å),^26^ it is 1.0 Å less than the experimental value. The predicted coherent fraction of unity for N in these nonadatom structures also compares particularly poorly with the experimental value of 0.43. Clearly, the comparison of the DFT and NIXSW results is not consistent with a structural model containing no Cu adatoms. Identifying which adatom models are most consistent with the experimental data is made more complex by the fact that these data are from a surface with coexisting α and β phases of unknown relative occupation.

**Table 2:** Predicted NIXSW Structural Parameters and Adsorption Energies Obtained from DFT Calculations of the Alternative Models of the α Phase of F_4_TCNQ on Cu(111)

	N		F		CF		CN		CC		
	*f*	*D* (Å)	*f*	*D* (Å)	*f*	*D* (Å)	*f*	*D* (Å)	*f*	*D* (Å)	adsorption energy (eV)
no adatom	0.99	2.13	0.91	3.44	0.98	3.44	0.99	2.64	0.90	3.22	–3.42
4 adatoms	0.02	2.60	0.98	3.36	0.98	3.35	0.65	2.88	0.95	3.23	–3.68
6 adatoms	0.54	2.85	0.92	3.34	0.97	3.32	0.84	2.95	0.96	3.20	–3.58
8 adatoms	1.00	2.89	0.98	3.30	0.99	3.26	1.00	2.99	0.98	3.16	–3.37
experimental value	0.43	3.11	0.43	3.31	0.87	3.33	0.60	3.15	0.64	3.32	

**Table 3:** Predicted NIXSW Structural Parameters and Adsorption Energies Obtained from DFT Calculations of the Alternative Models of the β Phase of F_4_TCNQ on Cu(111)

	N		F		CF		CN		CC		
	*f*	*D* (Å)	*f*	*D* (Å)	*f*	*D* (Å)	*f*	*D* (Å)	*f*	*D* (Å)	adsorption energy (eV)
no adatom	1.00	2.12	1.00	3.45	1.00	3.44	1.00	2.65	0.90	3.23	–3.52
4 adatoms	0.08	2.58	0.99	3.37	0.99	3.37	0.69	2.88	0.96	3.24	–3.67
6 adatoms	0.49	2.97	1.00	3.38	1.00	3.37	0.76	3.00	0.96	3.27	–3.94
8 adatoms	1.00	3.00	1.00	3.40	1.00	3.39	1.00	3.13	0.99	3.31	–3.95
experimental value	0.43	3.11	0.43	3.31	0.87	3.33	0.60	3.15	0.64	3.32	

For the β phase, the most energetically favored model has 8 adatoms. Notice that the locations of the Cu adatoms in this model coincide with the locations of weak protrusions in the STM image (Figure 2c), which could be due to the presence of Cu adatoms. The model also predicts NIXSW coherent positions that are generally in good agreement with the experimental values. The energetically favored α phase structure is the 4 adatom model. Notice that the predicted N coherent fractions for the 4 adatom models of both phases are very low; this can be attributed to the fact that half of the N atoms that are bonded to Cu adatoms are approximately 1.0 Å higher than the remaining N atoms that are bonded to the underlying Cu(111); this height difference is almost exactly one-half of the Cu(111) layer spacing, leading to a near-zero coherent fraction.^28^ Coexistence of these two lowest energy phases would lead to particularly good theory-experiment NIXSW parameter agreement, the combination of a high N coherent fraction in the β phase and a low coherent fraction in the α phase yielding a value similar to that of the experiment. Agreement with experimental and theoretical coherent positions for the N atoms would also be generally good for such a combination, as the value for the α phase will contribute only weakly due to the associated near-zero coherent fraction.

While the trends in these results are clear, the fact that the unit meshes of the overlayer phases are large and the number of Cu adatoms per unit mesh is unknown leads to a very large number of possible structural models. We have therefore performed DFT calculations for a range of additional models, chosen as described in the Supporting Information, and reported the energies and structural parameter values of these models in Tables S1 and S2. The conclusions are consistent with the results for the smaller number of alternative structures shown in Figure 5. In particular, the lowest-energy α and β phase structures have approximately 4 and 8 adatoms, respectively, while these structures have structural parameter values in best agreement with the NIXSW experimental data.

## Conclusions

Using a combination of NIXSW experiments and DFT calculations, we demonstrate that F_4_TCNQ adsorption on Cu(111) does lead to the incorporation of Cu adatoms into the molecular layer to form a 2D-MOF. Two phases were observed in our STM and LEED experiments, and though we cannot disentangle the relative contribution of the two phases to our NIXSW data, our DFT study does suggest that the α phase has one Cu adatom incorporated per F_4_TCNQ molecule, whereas the β phase has two Cu adatoms per molecule. Structural models with no adatoms in both phases lead to particularly poor agreement with the NIXSW data, especially for the N absorbers. Furthermore, the no adatom models of both phases are also significantly less energetically favorable than the alternative adatom models. The clear conclusion that a 2D-MOF is formed on Cu(111) by F_4_TCNQ adsorption is consistent with the pattern of behavior in F_4_TCNQ on Au(111)^21^ and Ag(100)^24^ and contrary to the conclusions of the previously published study of this adsorption system.^11^

## Supporting Information Available

Additional experimental results: LEED patterns during heating cycle; SXPS data: STM images of disordered regions produced by annealing to higher temperatures; NIXSW data; DFT calculations for additional structures (PDF)
