# Supplementary material for: Does F4TCNQ Adsorption on Cu(111) Form a 2D-MOF?
Source: J Phys Chem C Nanomater Interfaces. 2023 Oct 12;127(42):20903–10. doi: 10.1021/acs.jpcc.3c04927 (PMC10614301; doi:10.1021/acs.jpcc.3c04927)
Supplement: Supplementary file 1 — jp3c04927_si_001.pdf [file jp3c04927_si_001.pdf]

SUPPLEMENTARY INFORMATION

Does F_4TCNQ Adsorption on Cu(111) Form a 2D-MOF?

Pengcheng Ding[1,2], Mona Braim[1], A. L. Hobson[1,3], L.A. Rochford[3], P.T.P. Ryan[3,4] , D.A. Duncan[3], T.-L. Lee[3], H. Hussain[3], G. Costantini[5,6], Miao Yu[2], D.P. Woodruff[1]

(1) Department of Physics, University of Warwick, Coventry CV4 7AL, UK
(2) Laboratory for Space Environment and Physical Sciences, School of Chemistry and Chemical Engineering, Harbin Institute of Technology, Harbin 150001, China
(3) Diamond Light Source, Harwell Science and Innovation Campus, Didcot, OX11 0DE, UK
(4) Department of Materials, Imperial College, London SW7 2AZ, UK
(5) Department of Chemistry, University of Warwick, Coventry CV4 7AL, UK
(6) School of Chemistry, University of Birmingham, B15 2TT, UK

LEED patterns during heating cycle

Figure S1 shows the effect of heating and subsequent recooling on the LEED patterns from Cu(111)-F4TCNQ surface initially in a mixed α+β phase, transforming to an essentially single β phase . The values of the temperatures shown are as recorded in the experiments at the University of Warwick.

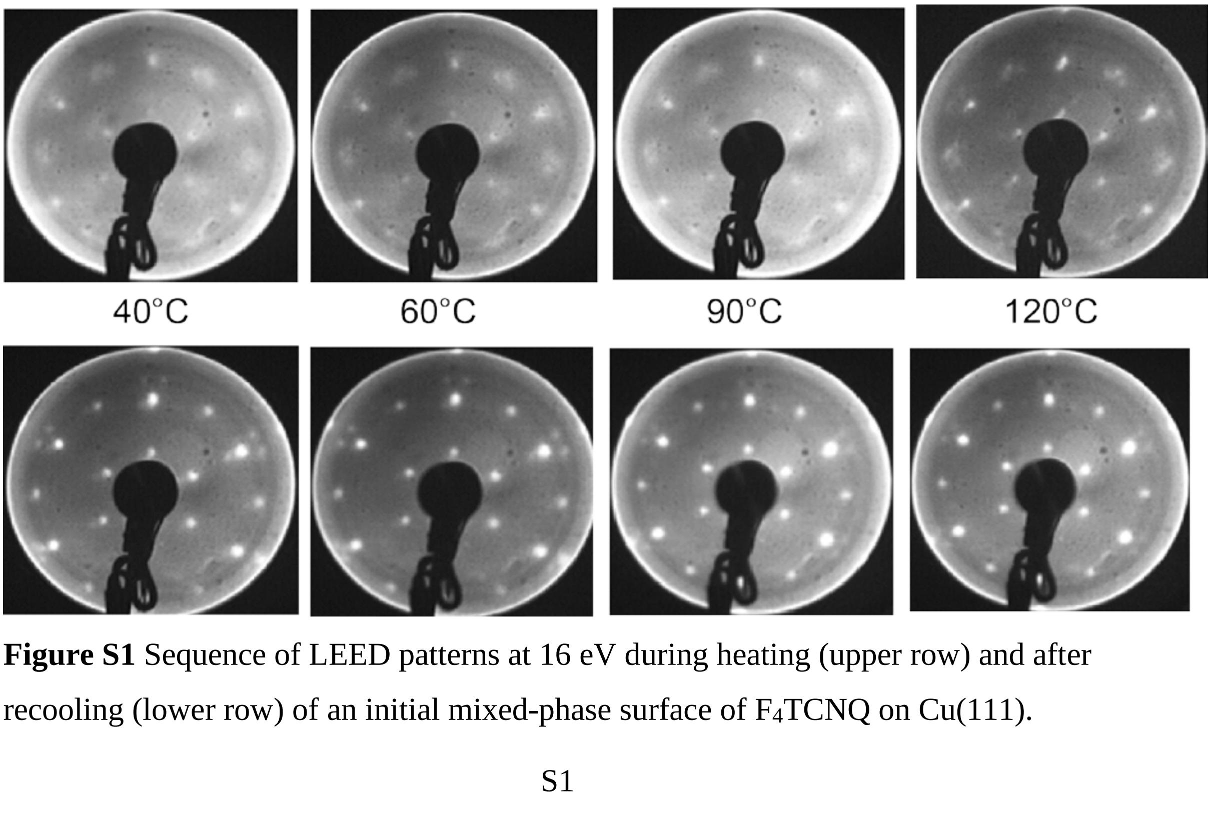

Figure S1 Sequence of LEED patterns at 16 eV during heating (upper row) and after recooling (lower row) of an initial mixed-phase surface of F_4TCNQ on Cu(111).

SXP spectra

The C 1s SXP spectrum from the as-prepared (weakly annealed) Cu(111)-F_4TCNQ surface is shown in Figure 4 of the main manuscript. N 1s and F 1s spectra from this surface, both exhibiting a single peak, are shown below in Figure S2.

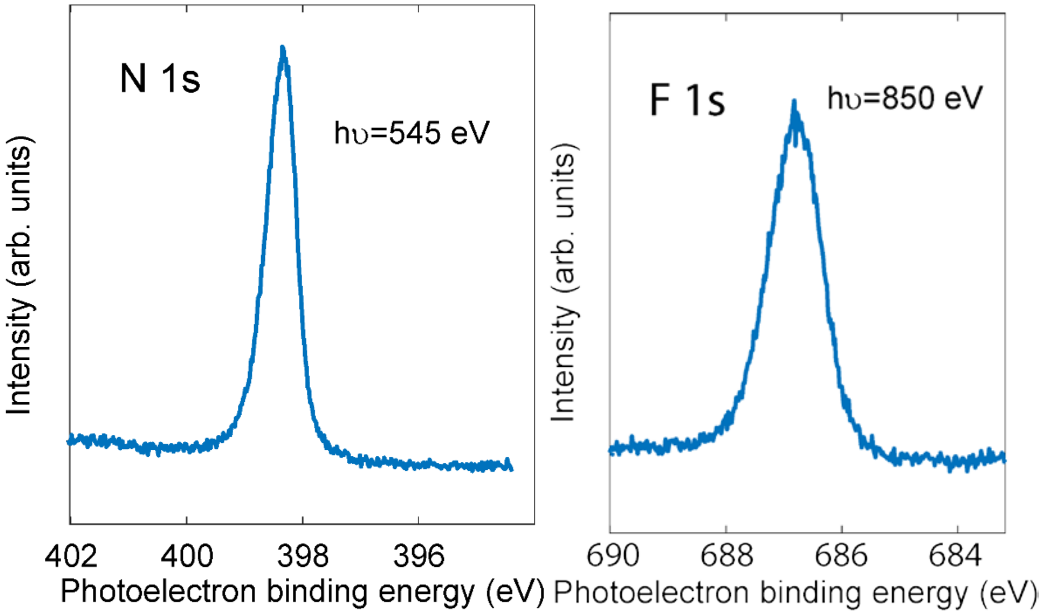

Figure S2. C 1s and N 1s SXP spectra from as-prepared (mixed phase) Cu(111)-F_4TCNQ surface

C 1s, N 1s and F 1s SXP spectra from the Cu(111)-F_4TCNQ sample annealed to a higher temperature to yield a LEED pattern characteristic of the β-phase are shown in Figure S3. The C 1s spectrum can be fitted by the same 4 components used to fit the spectrum from the as-prepared sample, but with the inclusion of at least 2 additional components. The weak peak attribute to atomic C from the as-prepared sample is greatly enhanced in amplitude after the higher temperature treatment. The exact assignment of the additional peaks is unknown. The N 1s spectrum from this surface shows only a single peak, although the width is larger than from the as-prepared sample, possibly indicative of more than one component. The F 1s spectrum after this heating shows a second well-resolved peak clearly indicating the presence of a second F species on the surface, as discussed in the main text.

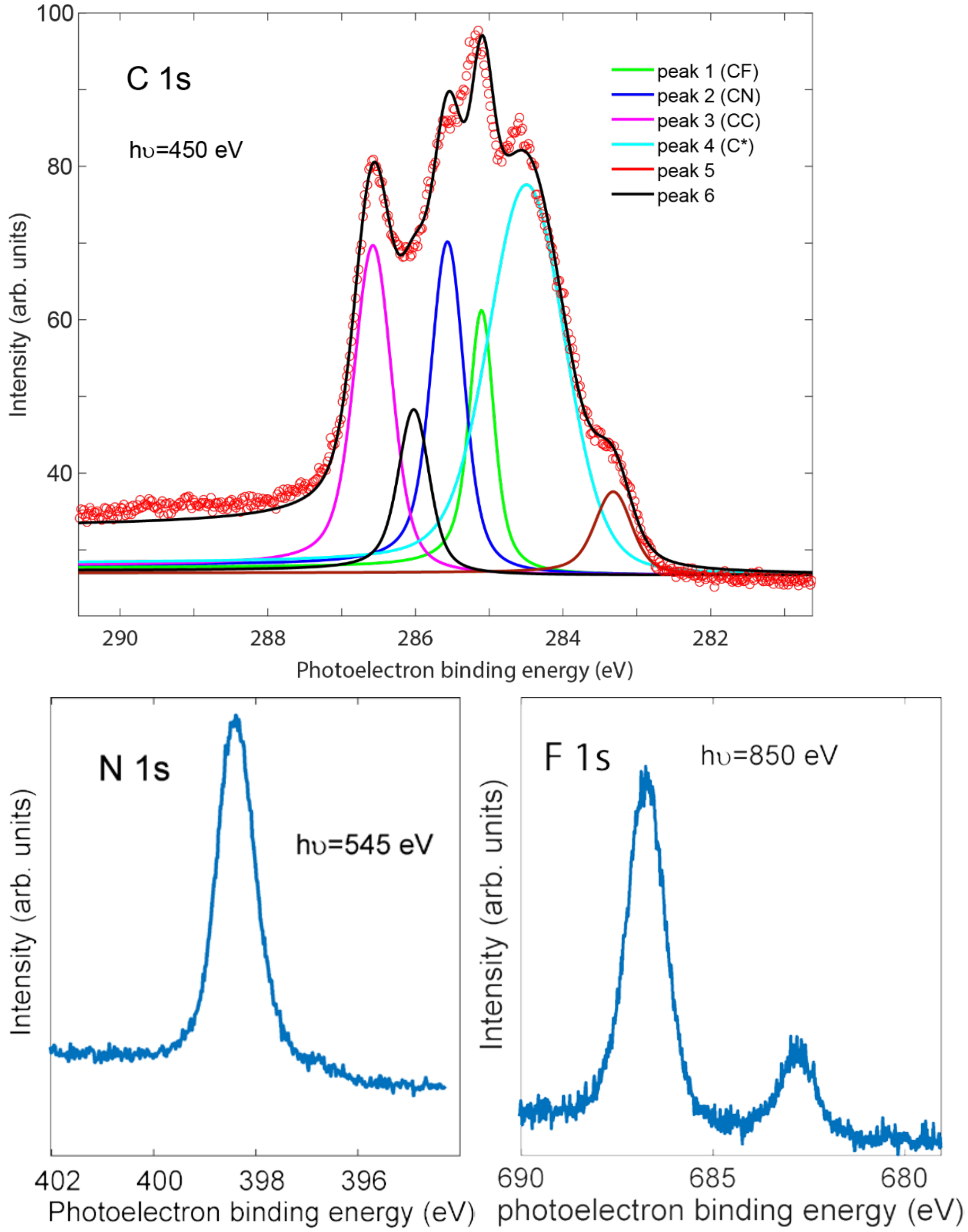

Figure S3. SXP spectra from annealed Cu(111)-F_4TCNQ surface

STM images showing the formation of disordered regions with annealing to increasingly higher temperatures.

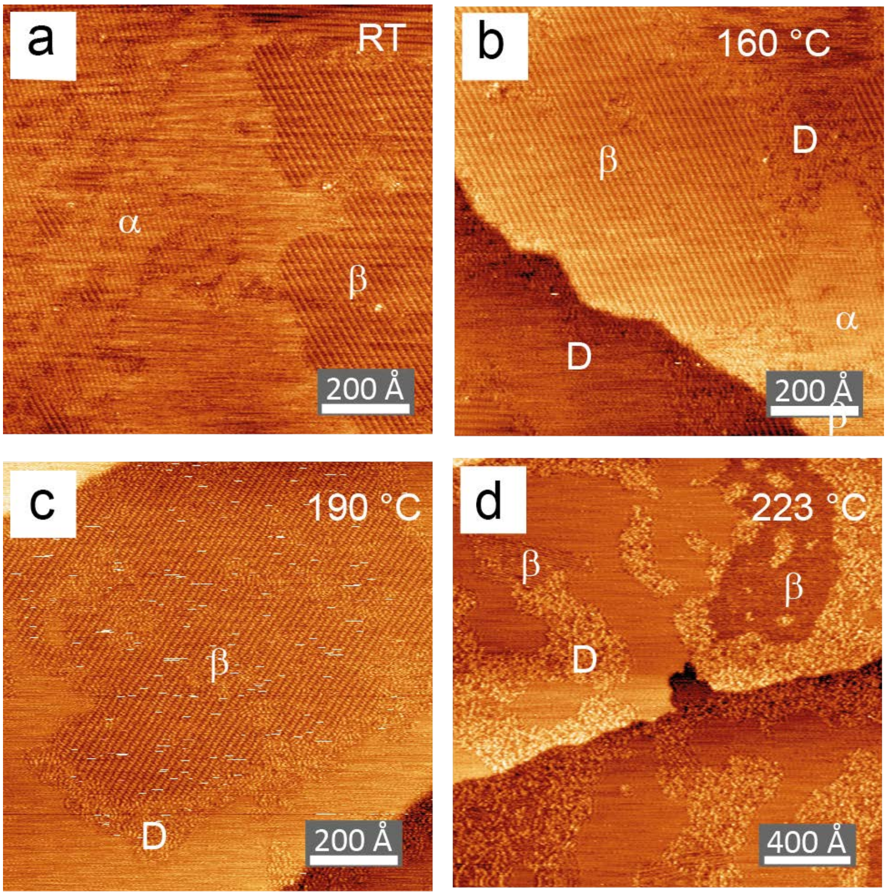

Figure S4 STM images of the Cu(111)-F_4TCNQ surface showing how annealing to successively higher temperatures after room temperature deposition not only leads initially to transformation of areas of α phase to β phase, but also the increasing appearance of disordered areas (labelled D).

NIXSW

Figure S5 shows the chemical-state resolved NIXSW data together with the fits having the coherent fraction and coherent position values listed in Table 1 of the main manuscript.

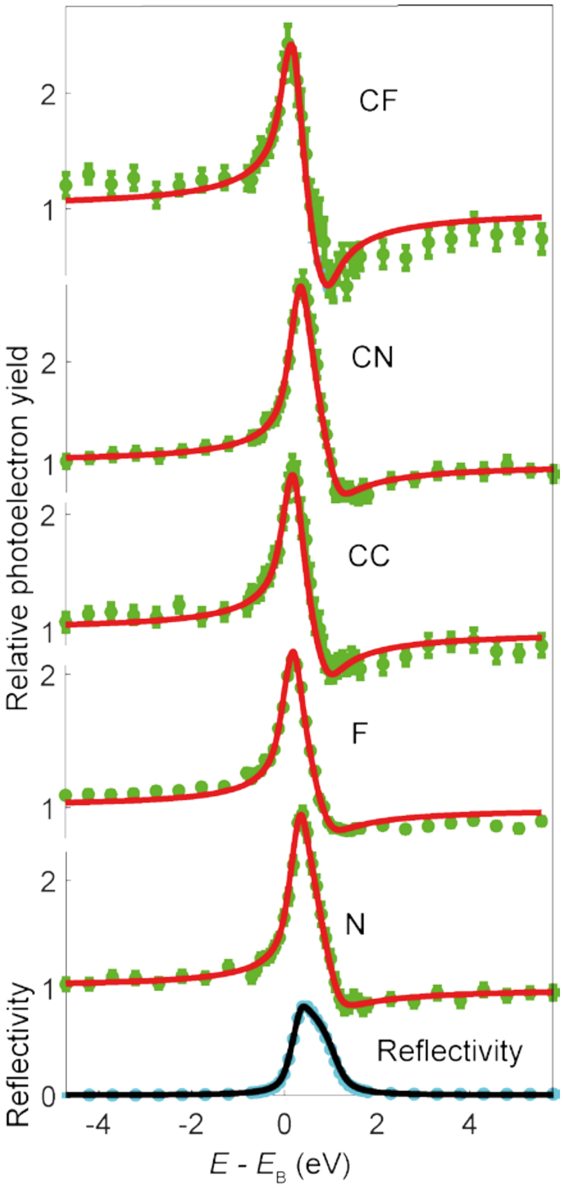

Figure S5. NIXSW experimental data points and superimposed fits (continuous lines); the corresponding values of *f* and *D* are given in Table 1.

DFT calculations for additional structures

Figure 5 of the main paper shows 4 alternative structural models for each of the α and β phases while the structural parameters of the lowest energy DFT structures of each of these models are presented in Tables 2 and 3. As a check on the generality of the conclusions drawn from these results we have extended the DFT calculations to a range of additional possible structures. Based on the experience of the studies of TCNQ and F_4TCNQ on Au and Ag surfaces in which adatoms are incorporated into the overlayers, we have started from the assumption that any Cu adatoms may be expected to occupy sites approximately midway between, cyano groups of adjacent molecules. These possible sites are shown numbered in Figure S5. This constraint still leads to a very large number of possible structures, but as both supercells consist of two columns of molecules it seems reasonable to assume the structure of adjacent rows are similar, consistent with the half-integer matrices found in both STM and LEED.

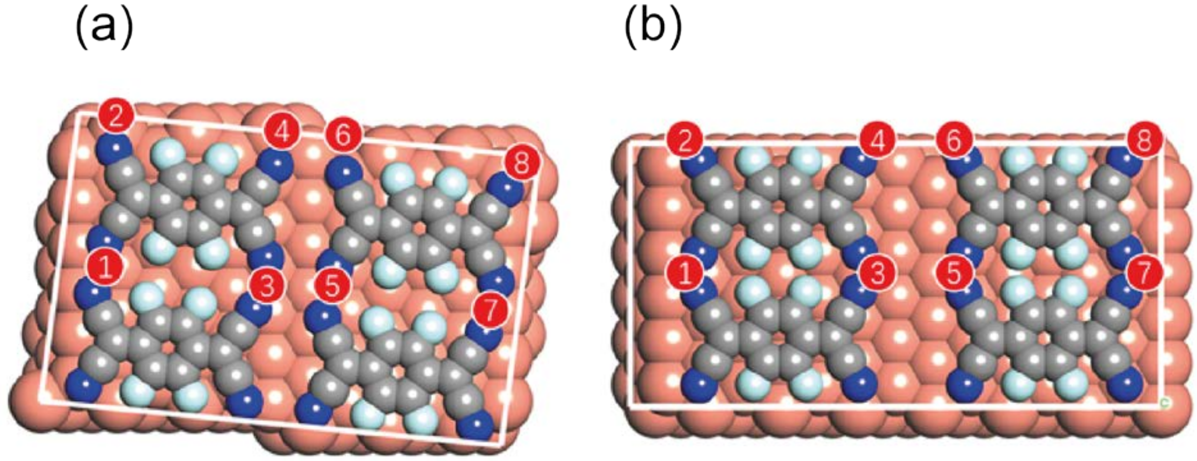

Figure S6. Models of the distribution of F_4TCNQ molecules in the α (a) and β (b) phases showing the possible Cu adatoms sites approximately midway between adjacent cyano groups.

Table S1 Results of DFT calculations for additional α-phase structural models with Cu adatoms in the numbered sites of Figure S5(a).

$\begin{pmatrix}12 & 5\\ 1 & 7\end{pmatrix}$	number of adatoms	N		F		CF		CN		CC		Adsorption energy (eV)
		f	*D*(Å)	*f*	*D*(Å)	*f*	*D*(Å)	*f*	*D*(Å)	*f*	*D*(Å)	
Adatom site occupation	0	0.99	2.13	0.91	3.44	0.98	3.44	0.99	2.64	0.90	3.22	–3.42
①	1	0.73	2.10	0.96	3.33	0.98	3.33	0.84	2.64	0.91	3.15	–3.40
①⑤	2	0.49	2.10	0.97	3.34	0.98	3.33	0.73	2.71	0.91	3.17	–3.47
①②⑤	3	0.39	2.29	0.66	3.37	0.88	3.38	0.79	2.75	0.89	3.20	–3.40
①④⑤	3	0.26	2.18	0.69	3.40	0.89	3.43	0.70	2.83	0.92	3.27	–3.62
①②⑤⑥	4	0.41	2.51	0.92	3.34	0.97	3.32	0.81	2.80	0.93	3.17	–3.28
①④⑤⑥	4	0.24	2.51	0.77	3.39	0.94	3.39	0.74	2.85	0.92	3.24	–3.57
①④⑤⑧	4	0.02	2.60	0.98	3.36	0.98	3.35	0.65	2.88	0.95	3.23	–3.68
①②④⑤⑥	5	0.41	2.71	0.78	3.35	0.94	3.34	0.81	2.88	0.95	3.19	–3.45
①②④⑤⑧	5	0.30	2.84	0.88	3.37	0.95	3.36	0.76	2.93	0.95	3.22	–3.65
①②④⑤⑥⑧	6	0.54	2.85	0.92	3.34	0.97	3.32	0.84	2.95	0.96	3.20	–3.58
①②③④⑤⑥⑧	7	0.76	2.88	0.88	3.33	0.96	3.29	0.92	2.97	0.97	3.18	–3.46
①②③④⑤⑥⑦⑧	8	1.00	2.89	0.98	3.30	0.99	3.26	1.00	2.99	0.98	3.16	–3.37
Experimental value		0.43	3.11	0.43	3.31	0.87	3.33	0.60	3.15	0.64	3.32	

Table S2 Results of DFT calculations for additional β-phase structural models with Cu adatoms in the numbered sites of Figure S5(b)

$\begin{pmatrix}7 & 14\\ 6 & 0\end{pmatrix}$	number of adatoms	N		F		CF		CN		CC		Adsorption energy (eV)
		f	*D*(Å)	*f*	*D*(Å)	*f*	*D*(Å)	*f*	*D*(Å)	*f*	*D*(Å)	
Adatom site occupation	0	1.00	2.12	1.00	3.45	1.00	3.44	1.00	2.65	0.90	3.23	–3.52
①	1	0.75	2.11	1.00	3.44	1.00	3.44	0.88	2.67	0.91	3.23	–3.61
①④	2	0.50	2.13	0.99	3.43	1.00	3.42	0.79	2.74	0.92	3.24	–3.70
①②④	3	0.31	2.24	0.97	3.44	0.99	3.44	0.75	2.80	0.92	3.26	–3.70
①④⑤	3	0.25	2.08	0.98	3.41	1.00	3.41	0.68	2.78	0.92	3.24	–3.79
①④⑤⑧	4	0.08	2.58	0.99	3.37	0.99	3.37	0.69	2.88	0.96	3.24	–3.67
①②④⑤⑧	5	0.24	3.01	0.99	3.37	0.99	3.36	0.67	2.93	0.95	3.24	–3.94
①②④⑤⑥⑧	6	0.49	2.97	1.00	3.38	1.00	3.37	0.76	3.00	0.96	3.27	–3.94
①②③④⑤⑥⑧	7	0.75	3.00	0.99	3.34	1.00	3.36	0.86	3.08	0.97	3.28	–3.94
①②③④⑤⑥⑦⑧	8	1.00	3.00	1.00	3.40	1.00	3.39	1.00	3.13	0.99	3.31	–3.95
Experimental value		0.43	3.11	0.43	3.31	0.87	3.33	0.60	3.15	0.64	3.32	
